# Successful endoscopic ultrasound-guided fine-needle biopsy of a recurrent paraganglioma using a forward-viewing echoendoscope in a patient who had undergone the Whipple procedure

**DOI:** 10.1055/a-2467-3509

**Published:** 2024-12-17

**Authors:** Miki Wada, Yukitoshi Matsunami, Shuntaro Mukai, Atsushi Sofuni, Takayoshi Tsuchiya, Yuichi Nagakawa, Takao Itoi

**Affiliations:** 1 13112 Department of Gastroenterology and Hepatology, Tokyo Medical University, Shinjuku-ku, Japan; 2 Department of Gastrointestinal and Pediatric Surgery, Tokyo Medical University, Shinjuku-ku, Japan

Endoscopic ultrasound-guided fine-needle biopsy (EUS-FNB) is useful for the diagnosis of retroperitoneal lesions and pancreatic diseases. However, the usefulness of EUS-FNB for tissue acquisition from retroperitoneal lesions in patients with surgically altered anatomy has not been established 1 2 3 4 5 . Herein, we report successful tissue acquisition from a recurrent lymph node lesion of a retroperitoneal paraganglioma by EUS-FNB using a forward-viewing echoendoscope (FV-EUS) in a patient who had undergone the Whipple procedure.

A 60-year-old man underwent the Whipple procedure for a retroperitoneal paraganglioma adjacent to the head of the pancreas ( Fig. 1 ). Follow-up computed tomography 2.5 years after surgery revealed a 15-mm swelling of the lymph node on the right side of the inferior vena cava ( Fig. 2 ). The lesion was located near the afferent loop, and we expected that it could be visualized using an FV-EUS (TGF-UC260J; Olympus, Tokyo, Japan).

**Fig. 1:**
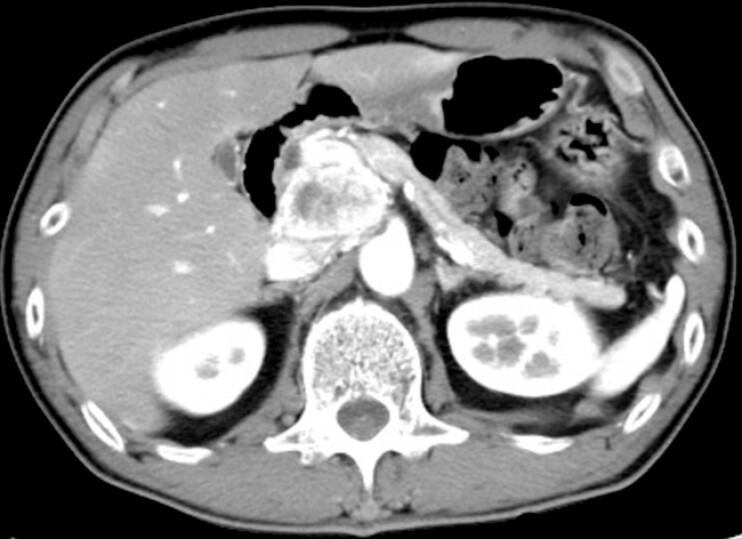
Computed tomography image of a retroperitoneal paraganglioma. The tumor was located adjacent to the head of the pancreas.

**Fig. 2:**
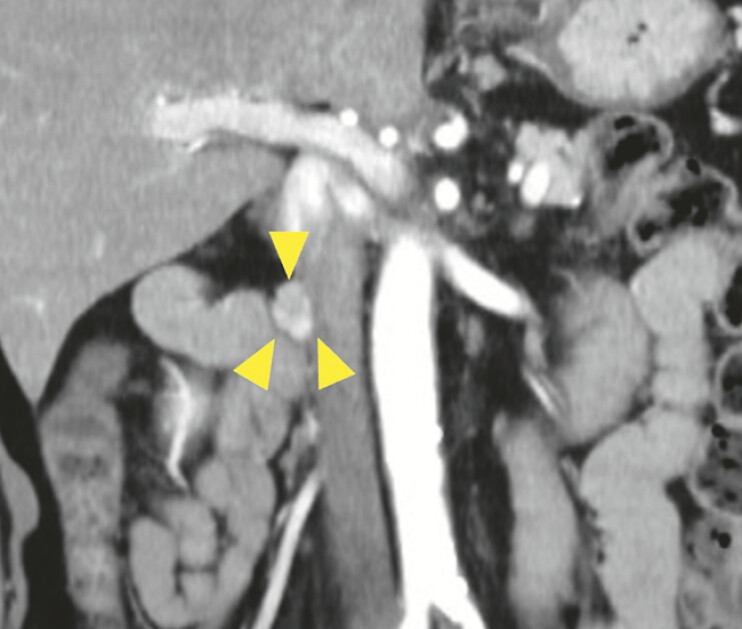
Computed tomography image of a recurrent lymph node lesion (arrowheads) of the retroperitoneal paraganglioma.

To insert the FV-EUS into the afferent loop safely, a short-type single-balloon enteroscope (SIF-H290; Olympus) was first inserted into the hepaticojejunostomy anastomosis ( Fig. 3 ), and a guidewire was placed. Then, under wire guidance, the FV-EUS was inserted up into the afferent loop, and the target lesion was visualized. EUS-FNB was performed transjejunally using a 22-gauge FNB needle ( Fig. 4 ). The histopathological diagnosis was consistent with lymph node recurrence of the retroperitoneal paraganglioma ( Fig. 5 ). Finally, open retroperitoneal tumor resection was performed, and complete resection was achieved ( Video 1 ).

**Fig. 3:**
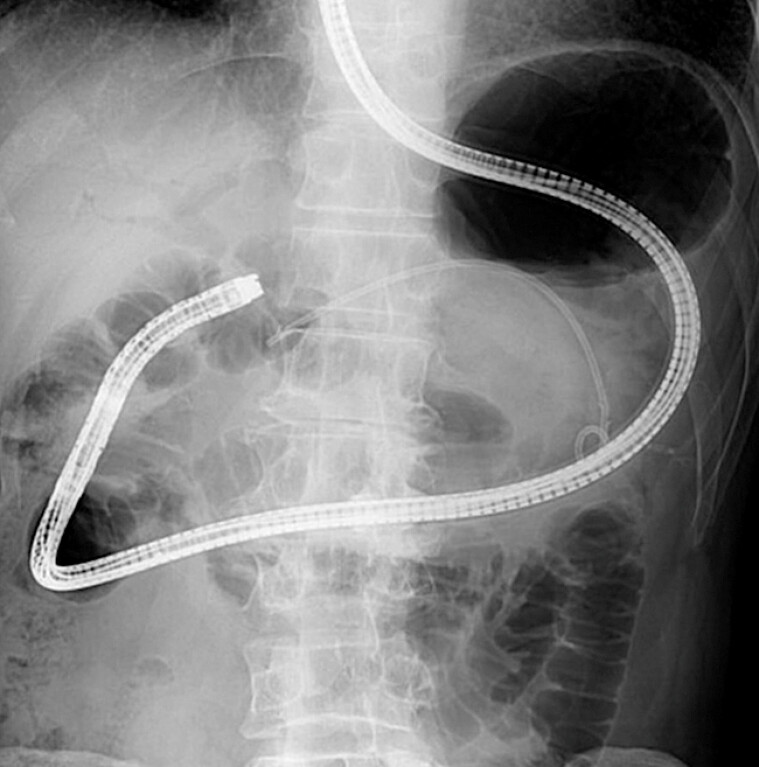
A balloon enteroscope was inserted into the afferent loop.

**Fig. 4:**
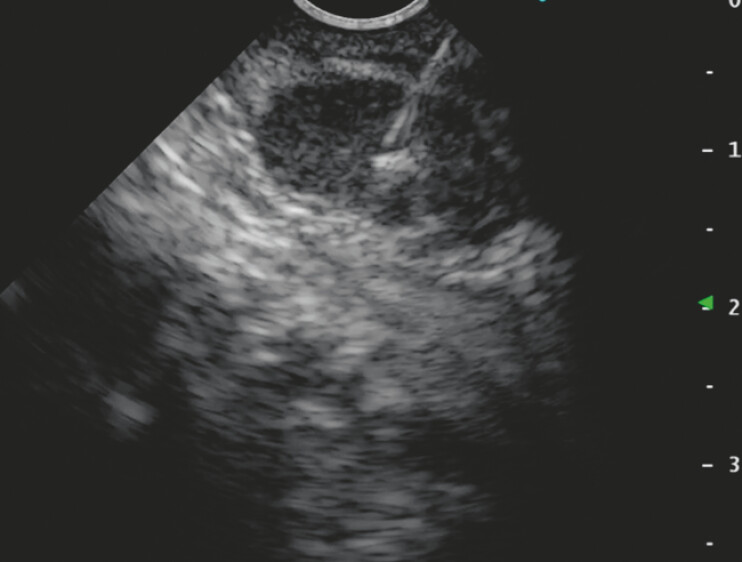
Endoscopic ultrasound-guided fine-needle biopsy was performed using a 22-gauge needle.

**Fig. 5:**
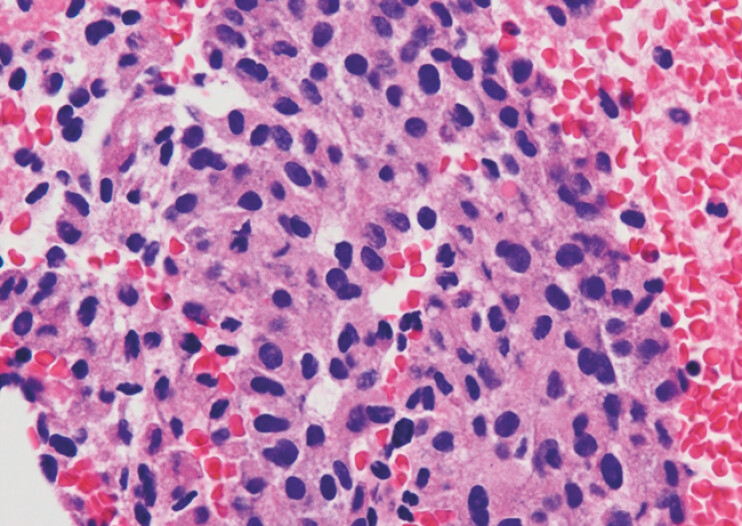
The pathological specimen obtained by endoscopic ultrasound-guided fine-needle biopsy.

Successful endoscopic ultrasound-guided fine-needle biopsy of a recurrent paraganglioma using a forward-viewing echoendoscope in a patient who had undergone the Whipple procedure.Video 1

This case demonstrates that EUS-FNB using an FV-EUS and assisted by balloon enteroscope insertion is a safe and effective method for tissue acquisition in patients with surgically altered anatomy, and can prevent adverse events, such as gastrointestinal perforation.

Endoscopy_UCTN_Code_TTT_1AS_2AC

## Notes

Endoscopy E-Videos https://eref.thieme.de/e-videos
*E-Videos* is an open access online section of the journal *Endoscopy* , reporting on interesting cases and new techniques in gastroenterological endoscopy. All papers include a high-quality video and are published with a Creative Commons CC-BY license. Endoscopy E-Videos qualify for HINARI discounts and waivers and eligibility is automatically checked during the submission process. We grant 100% waivers to articles whose corresponding authors are based in Group A countries and 50% waivers to those who are based in Group B countries as classified by Research4Life (see: https://www.research4life.org/access/eligibility/ ). This section has its own submission website at https://mc.manuscriptcentral.com/e-videos .

## References

[1] Katanuma A Hayashi T Kin T Interventional endoscopic ultrasonography in patients with surgically altered anatomy: techniques and literature review Dig Endosc 2020 32 263 274 31643105 10.1111/den.13567

[2] Larghi A Fuccio L Chiarello G Fine-needle tissue acquisition from subepithelial lesions using a forward-viewing linear echoendoscope Endoscopy 2014 46 39 45 24218311 10.1055/s-0033-1344895

[3] Tanaka K Hayashi T Utsunomiya R Endoscopic ultrasound-guided fine needle aspiration for diagnosing pancreatic mass in patients with surgically altered upper gastrointestinal anatomy Dig Endosc 2020 32 967 973 31912558 10.1111/den.13625

[4] Gong TT Zhang MM Zou DW EUS-FNA of a lesion in the pancreatic head using a forward-viewing echoendoscope in a patient with Billroth II gastrectomy (with video) Endosc Ultrasound 2022 11 243 245 35017384 10.4103/EUS-D-21-00101 PMC9258016

[5] Akdamar MK Eltoum I Eloubeidi MA Retroperitoneal paraganglioma: EUS appearance and risk associated with EUS-guided FNA Gastrointest Endosc 2004 60 1018 1021 10.1016/s0016-5107(04)02218-7 15605027

